# Diffusion Maximum Correntropy Criterion Based Robust Spectrum Sensing in Non-Gaussian Noise Environments

**DOI:** 10.3390/e20040246

**Published:** 2018-04-03

**Authors:** Xiguang Xu, Hua Qu, Jihong Zhao, Feiyu Yan, Weihua Wang

**Affiliations:** 1 School of Electronic and Information Engineering, Xi’an Jiaotong University, Xi’an 710049, China; 2 Suzhou Caiyun Network Technologies Co., Ltd., Suzou 215123, China; 3 School of Telecommunication and Information Engineering, Xi’an University of Posts and Telecommunications, Xi’an 710061, China

**Keywords:** robust spectrum sensing, maximum correntropy criterion (MCC), diffusion scheme, non-Gaussian noise, cognitive radio networks

## Abstract

Spectrum sensing is the most important task in cognitive radio (CR). In this paper, a new robust distributed spectrum sensing approach, called diffusion maximum correntropy criterion (DMCC)-based robust spectrum sensing, is proposed for CR in the presence of non-Gaussian noise or impulsive noise. The proposed distributed scheme, which does not need any central processing unit, is characterized by an adaptive diffusion model. The maximum correntropy criterion, which is insensitive to impulsive interference, is introduced to deal with the effect of non-Gaussian noise. Simulation results show that the DMCC-based spectrum sensing algorithm has an excellent robust property with respect to non-Gaussian noise. It is also observed that the new method displays a considerably better detection performance than its predecessor (i.e., diffusion least mean square (DLMS)) in impulsive noise. Moreover, the mean and variance convergence analysis of the proposed algorithm are also carried out.

## 1. Introduction

Cognitive radio (CR), which can exploit the vacant spectrum dynamically, has been considered as a promising technology for response spectrum scarcity [1,2,3]. The most important component of CR is spectrum sensing. This is because cognitive users (CUs) should know their own operating environments to make sure that the primary users (PUs) are not interfered with by CUs. A large number of schemes has been proposed for this target, including energy detection [4], matched filtering detection [5], cyclostationary detection [6] and cooperative spectrum sensing [7,8].

Cooperative spectrum sensing is proposed to tackle the problem of shadow fading and hidden primary receivers [9]. Among the cooperative spectrum sensing methods, centralized cooperative sensing [9,10] lets each CU send information to a central processing unit, and then, the decisions about the presence or absence of the PU are made by a certain fusion algorithm. In [11], fuzzy data fusion Kalman filter-based cooperative spectrum sensing has been proposed to make a global sensing decision. The fuzzy data fusion Kalman filter, which has been applied to reduce failure risk in an integrated vehicle health maintenance system (IVHMS) [12], is an effective and reliable method to improve sensing performance in CR. Because of the central processing unit, high communication overhead and enormous computing power are needed [13]. To solve these problems, decentralized cooperative sensing methods have been proposed, such as consensus algorithms [14,15], belief propagation [16] and diffusion methods [17,18,19].

Some recent literature [17,18,19] has proposed distributed diffusion methods (diffusion least mean square (DLMS)), which allow each CU to collect data from its neighbors and make detection decisions based on these data without any central processing unit. As it is a distributed and adaptive diffusion scheme, DLMS has an ability to enhance network failure resistance. However, this diffusion solution is only suitable for Gaussian noise. If the network is disturbed by non-Gaussian noise or so-called impulsive noise, the detection performance of this diffusion solution may deteriorate seriously.

Typical non-Gaussian noise distribution [20,21,22,23] has heavy tails, which may be generated by multiple impulsive interference. For example, the impulsive nature of the noise in industrial, scientific and medical (ISM) bands leads to impulsive noise. Impulsive noise is also caused by microwave ovens or devices with electromechanical switches. In addition, impulsive interference is also caused by various components on a computer platform. What is more, igniting car engines, power lines and heavy current switches in urban environments are the typical man-made impulsive noise sources. Impulsive interference in real-world environments degrades the performance of spectrum sensing.

In order to solve the non-Gaussian noise problem in CR, various robust spectrum sensing approaches have been published [24,25,26,27] in recent years. In [24], a *p*-th order moment-based spectrum sensing has been proposed to counteract impulsive noise. An accurate kernelized energy detector is displayed in [25]. Besides, Ref. [26] presents a soft-limited polarity-coincidence-array spectrum sensing to detect the primary signal distorted by non-Gaussian noise. However, these approaches are single-user detections, which have a limited capability to detect the signal in complex noise environments. In [27], a multi-user detection algorithm called Rao test-based cooperative spectrum sensing has been proposed for robust detection. Although this detection scheme performs well in centralized cooperation, it needs a central processing unit. The disadvantage of the central unit is obvious, that is once the central unit fails, it can easily lead to paralysis of the entire network. In order to solve the shortcoming of centralized cooperation and improve robustness for CR, a robust distributed cooperation spectrum sensing approach is needed. As far as we know, there are no previous studies in the literature dealing with the robust distributed spectrum sensing in non-Gaussian noise environments.

In this paper, a distributed robust spectrum sensing (without any central processing unit), called diffusion maximum correntropy criterion (DMCC)-based robust spectrum sensing, is proposed in non-Gaussian noise environments. The correntropy, as a nonlinear similarity measure in information theoretic learning (ITL), has been successfully used in non-Gaussian noise for its robust and cost-efficient function [28,29,30]. The new distributed robust spectrum sensing is motivated by the desirable features of correntropy and the diffusion model schemes [17,18]. The main contributions of this paper are three-fold: A DMCC-based robust spectrum sensing scheme is presented to solve the distributed power estimation with non-Gaussian noise; a version of DMCC-based robust spectrum sensing, namely adaptation to combination DMCC (ATC DMCC) algorithm, is derived, which can solve the non-Gaussian noise problem in spectrum sensing; the mean and mean square performance of the new power estimation algorithm have been analyzed. In addition, the simulation results show that the performance of the proposed method is excellent under impulsive noise environments.

This paper is organized as follows: in Section 2, we describe the signal model and non-Gaussian noise model; a brief review of MCC is given in Section 3; in Section 4, we derive the distributed DMCC-based power estimation algorithm and develop the DMCC-based robust spectrum sensing algorithm; in Section 5, we present the mean analysis, the variance convergence analysis and the detection performance analysis; in Section 6, the performance of the proposed sensing scheme is evaluated and compared with existing sensing algorithms. Finally, the conclusion is given in Section 7.

## 2. Signal Model and Non-Gaussian Noise Model

### 2.1. Signal Model

In this paper, every CU is interested in performing spectrum sensing in a distributed manner without any central processing unit, where CU gets the available information from its neighbors. It is assumed that the information transfer between neighbor CUs is lossless. Generally, the spectrum sensing problem in a distributed system can be described as a two-hypotheses decision as:

(1) $$\begin{array}{c} \left\{\begin{array}{c} H_{0}:x_{k}\left(n\right)=v_{k}\left(n\right)\\ H_{1}:x_{k}\left(n\right)=a_{k}s\left(n\right)+v_{k}\left(n\right),\left(n=1,2,\ldots \right) \end{array}\right. \end{array}$$

where k=1,2,…,K is the CU number and n=1,2,…,N is the sample index. $x_{k}\left(n\right)$ is the received signal of the CU *k*; $s\left(n\right)$ is the signal emitted by the PU; $v_{k}\left(n\right)$ can be regarded as the non-Gaussian noise, which will be described in detail below. $s\left(n\right)$ and $v_{k}\left(n\right)$ both are independently and identically distributed (i.i.d). At the same time, they are statistically independent of each other. $a_{k}$ represents the channel gain of the CU *k*. As the channel is assumed to be a slowly fading channel in this paper, the channel gain can be considered constant and obtained by: $a_{k}∼N\left(0,1\right)$. The two-hypotheses $H_{0}$ and $H_{1}$ denote the absence and presence of the PU signal, respectively.

In energy detection theory [4], an energy detector is an energy measurer that gauges the energy of the received signal. Based on these energy data, it decides whether the received waveform contains the PU signal. According to (1), we obtain the energy model at CU *k*:

(2) $$\begin{array}{c} \left\{\begin{array}{c} H_{0}:E\left[{\left|x_{k}\left(n\right)\right|}^{2}\right]=E\left[{\left|v_{k}\left(n\right)\right|}^{2}\right]\\ H_{1}:E\left[{\left|x_{k}\left(n\right)\right|}^{2}\right]=E\left[{\left|a_{k}\right|}^{2}{\left|s\left(n\right)\right|}^{2}\right]+E\left[{\left|v_{k}\left(n\right)\right|}^{2}\right] \end{array}\right. \end{array}$$

where $E\left[{\left|x_{k}\left(n\right)\right|}^{2}\right]$ denotes the average power of the received data samples, $E\left[{\left|v_{k}\left(n\right)\right|}^{2}\right]=\sigma_{v}^{2}$ is the average power of noise and the power of the PU signal is $E\left[{\left|s\left(n\right)\right|}^{2}\right]=S$. In this paper, the received power estimated by CU *k* is represented by $P_{k}\left(n\right)$.

### 2.2. Non-Gaussian Noise Model

The non-Gaussian noise, which is also called impulsive noise, is modeled as a Gaussian mixture in this paper. The Gaussian mixture model has been widely applied in wireless communications. We can get the impulsive noise by [31]:

(3) $$\begin{array}{c} v_{k}\left(n\right)=g_{1,k}\left(n\right)+d_{k}\left(n\right)g_{2,k}\left(n\right) \end{array}$$

where $g_{1,k}\left(n\right)$ and $g_{2,k}\left(n\right)$, the two zero mean Gaussian noises with variances $\sigma_{1}^{2}$ and $\sigma_{2}^{2}$, respectively, are independent. $d_{k}\left(n\right)$, a sequence of ones and zeros, which is an independently and identically distributed (i.i.d) Bernoulli random process with occurrence probabilities, $P_{r}\left(d_{k}\left(n\right)=1\right)=p$. It is necessary to note that the variance $\sigma_{2}^{2}$ is chosen to be much larger than $\sigma_{1}^{2}$, so that a large impulse will appear when $d_{k}\left(n\right)=1$.

## 3. Brief Background of the Maximum Correntropy Criterion

MCC has been successfully and widely applied in adaptive filtering [29,30]. Correntropy is generalized to measure the similarity of two random variables. The correntropy is defined as [28]:

(4) $$\begin{array}{c} V\left(X,Y\right)=E\left[\kappa \left(X,Y\right)\right]=\int \kappa \left(x,y\right)dF_{x,y}\left(x,y\right) \end{array}$$

where *E* denotes the expectation operator, $F_{x,y}\left(x,y\right)$ is the joint distribution of the two variables and $\kappa \left(\cdot ,\cdot \right)$ is a Mercer kernel. In practice, the joint distribution $F_{x,y}\left(x,y\right)$ is unavailable, and the number of data we know is limited. In these cases, the correntropy can be estimated as the sample mean:

(5) $$\begin{array}{c} V\left(X,Y\right)=E\left[\kappa \left(X,Y\right)\right]\approx \frac{1}{N}\sum_{i=1}^{N}\kappa \left(x_{i},y_{i}\right) \end{array}$$

where *N* is the sample number.

In this paper, the most popular Gaussian kernel [32] is applied in correntropy, and it can be expressed as:

(6) $$\begin{array}{c} \kappa \left(x,y\right)=\frac{1}{\sqrt{2\pi }\sigma }\exp\left(-\frac{e^{2}}{2\sigma^{2}}\right) \end{array}$$

where e=x−y is the error and σ is the kernel size. According to the Gaussian kernel, the instantaneous MCC cost is given by [29]:

(7) $$\begin{array}{c} J_{MCC}\left(n\right)=\frac{1}{\sqrt{2\pi }\sigma }\exp\left(\frac{-e^{2}\left(n\right)}{2\sigma^{2}}\right) \end{array}$$

MCC has some desirable advantages. For example, it is almost bounded for any distribution; it is also a local similarity measure and is robust to outliers. Based on these favorable advantages, we derive the distributed diffusion MCC-based power estimation algorithm in the following section.

## 4. Distributed Diffusion Maximum Correntropy Criterion-Based Power Estimation and Spectrum Detection

### 4.1. Derivation of the Distributed DMCC-Based Power Estimation Algorithm

According to the energy model (2), each CU receives the signal transmitted by the PU and estimates its power. As the channel gain is different, the estimated power at each CU differs. When the channel condition at CU *k* is poor, the power is low. On the contrary, if the CU has a good channel condition, it will have a high power performance. In this article, CUs cooperate to estimate a common parameter $P^{o}$, the average power of all CUs.

(8) $$\begin{array}{c} P^{o}=\frac{1}{K}\sum_{k=1}^{K}E\left[{\left|x_{k}\left(n\right)\right|}^{2}\right]=S\frac{1}{K}\sum_{k=1}{\left|a_{k}\right|}^{2}+\sigma_{v}^{2} \end{array}$$

According to the MCC cost (7), the global cost function for each CU can be expressed as:

(9) $$\begin{array}{c} J^{glob}\left(P\right)=\sum_{k=1}^{K}\frac{1}{\sqrt{2\pi }\sigma }\exp\left(-\frac{1}{2\sigma^{2}}{\left|{\left|x_{k}\left(n\right)\right|}^{2}-P\right|}^{2}\right) \end{array}$$

The optimal solution is obtained by minimizing (9).

In this paper, every CU is interested in estimating the average power $P^{o}$ in a distributed manner, where the CU gets the available information from its neighbors. The distributed manner does not need any central processing unit, which improves the robustness and stability of the algorithm. The local cost function [33] of the DMCC for each CU is defined as:

(10) $$\begin{array}{c} J_{k}^{loc}\left(P\right)=\sum_{l\in N_{k}}\alpha_{l,k}\frac{1}{\sqrt{2\pi }\sigma }\exp\left(-\frac{1}{2\sigma^{2}}{\left|{\left|x_{l}\left(n\right)\right|}^{2}-P\right|}^{2}\right) \end{array}$$

where $N_{k}$ is the neighborhood set of CU *k*, $\left\{\alpha_{l,k}\right\}$ is a set of nonnegative coefficients, which satisfy the following conditions:

(11) $$\begin{array}{c} \alpha_{l,k}\geq 0,\sum_{l=1}^{K}\alpha_{l,k}=1,and\;\alpha_{l,k}=0\;if\;l\notin N_{k} \end{array}$$

This means that for every CU *k*, the sum of the coefficients $\left\{\alpha_{l,k}\right\}$ is one. We collect the entries $\left\{\alpha_{l,k}\right\}$ into a K×K matrix A.

The derivative of (10) is:

(12) $$\begin{array}{c} \nabla J_{k}^{loc}\left(P\right)=\frac{1}{\sigma^{2}}\sum_{l\in N_{k}}\alpha_{l,k}\frac{1}{\sqrt{2\pi }\sigma }\exp\left(-\frac{1}{2\sigma^{2}}{\left|{\left|x_{l}\left(n\right)\right|}^{2}-P\right|}^{2}\right)\left({\left|x_{l}\left(n\right)\right|}^{2}-P\right) \end{array}$$

We take the steepest descent method to yield:

(13) $$\begin{array}{cc} P_{k}\left(n+1\right) & =P_{k}\left(n\right)+\eta_{k}\nabla J_{k}^{loc}\left(P\right)\\ & =P_{k}\left(n\right)+\frac{\eta_{k}}{\sigma^{2}}\sum_{l\in N_{k}}\alpha_{l,k}\frac{1}{\sqrt{2\pi }\sigma }\exp\left(-\frac{1}{2\sigma^{2}}{\left|{\left|x_{l}\left(n\right)\right|}^{2}-P\right|}^{2}\right)\left({\left|x_{l}\left(n\right)\right|}^{2}-P\right) \end{array}$$

where $\eta_{k}$ is the step size. For the sake of simplicity, we set $\mu_{k}=\frac{\eta_{k}}{\sqrt{2\pi }\sigma^{3}}\left(k=1,2,\cdots ,K\right)$ as the new step size. Therefore we have:

(14) $$\begin{array}{c} P_{k}\left(n+1\right)=P_{k}\left(n\right)+\mu_{k}\sum_{l\in N_{k}}\alpha_{l,k}\exp\left(-\frac{1}{2\sigma^{2}}{\left|{\left|x_{l}\left(n\right)\right|}^{2}-P\right|}^{2}\right)\left({\left|x_{l}\left(n\right)\right|}^{2}-P\right) \end{array}$$

Next, we can obtain the intermediate estimates of each CU by:

(15) $$\begin{array}{c} \psi_{k}\left(n\right)=\sum_{l\in N_{k}}\beta_{l,k}P_{l}\left(n\right) \end{array}$$

where $\psi_{k}\left(n\right)$ represents an intermediate estimate for CU *k* at instant *n*. The non-negative element $\beta_{l,k}$ defines if the estimate from CU *l* (including CU *k*) is available for CU *k*. They satisfy the conditions:

(16) $$\begin{array}{c} \beta_{l,k}\geq 0,\sum_{l=1}^{K}\beta_{l,k}=1,and\;\beta_{l,k}=0\;if\;l\notin N_{k} \end{array}$$

We collect the entries $\left\{\beta_{l,k}\right\}$ into a K×K matrix B. With the intermediate estimates, the CUs update the estimates by (14):

(17) $$\begin{array}{c} \theta_{k}\left(n\right)=\psi_{k}\left(n\right)+\mu_{k}\sum_{l\in N_{k}}\alpha_{l,k}\exp\left(-\frac{1}{2\sigma^{2}}{\left|{\left|x_{l}\left(n\right)\right|}^{2}-\psi_{k}\left(n\right)\right|}^{2}\right)\left({\left|x_{l}\left(n\right)\right|}^{2}-\psi_{k}\left(n\right)\right) \end{array}$$

The coefficients $\left\{\alpha_{l,k}\right\}$ decide which CUs should share their measurements with CU *k*. At last, each CU combines the estimates as:

(18) $$\begin{array}{c} P_{k}\left(n+1\right)=\sum_{l\in N_{k}}\gamma_{l,k}\theta_{l}\left(n\right) \end{array}$$

where the coefficients $\left\{\gamma_{l,k}\right\}$ are similar to $\left\{\beta_{l,k}\right\}$, and they represent whether CUs should share their intermediate estimates $\theta_{l}\left(n\right)$ with CU *k*. We collect the entries $\left\{\gamma_{l,k}\right\}$ into a K×K matrix R.

There are detailed descriptions of the selection of the weights $\beta_{l,k}$, $\alpha_{l,k}$ and $\gamma_{l,k}$ in [34]. We can see that Equation (17) is similar to those in [33,34]. The only difference is that it contains an extra scaling factor $\exp\left(-\frac{1}{2\sigma^{2}}{\left|{\left|x_{l}\left(n\right)\right|}^{2}-\psi_{k}\left(n\right)\right|}^{2}\right)$, which is an exponential function of the error. When a large noise occurs, this factor is close to zero, which endows the DMCC method with robustness and significantly improves the adaptation performance in impulsive noise.

### 4.2. ATC DMCC-Based Power Estimation

There are mainly two different schemes (including the adapt-then-combine (ATC) scheme and the combine-then-adapt (CTA) scheme) for the diffusion estimation [33,34]. The ATC scheme first utilizes the adaptive algorithm to update the local estimates and then combines the estimates. The CTA scheme, however, has a reverse order. As the learning performances of the two versions of DMCC-based algorithms are almost the same [34], we only discuss the ATC DMCC in this paper.

According to the adapt-then-combine scheme, one can obtain the following ATC DMCC method for the power estimation by combining (17) and (18):

(19) $$\left\{\begin{array}{c} \theta_{k}\left(n\right)=P_{k}\left(n\right)+\mu_{k}\sum_{l\in N_{k}}\alpha_{l,k}\exp\left(-\frac{1}{2\sigma^{2}}{\left|{\left|x_{l}\left(n\right)\right|}^{2}-P_{k}\left(n\right)\right|}^{2}\right)\left({\left|x_{l}\left(n\right)\right|}^{2}-P_{k}\left(n\right)\right)\\ P_{k}\left(n+1\right)=\sum_{l\in N_{k}}\gamma_{l,k}\theta_{l}\left(n\right) \end{array}\right.$$

The ATC DMCC estimation algorithm consists of two parts, an information exchange step and a combination step. In the information exchange step, every CU utilizes the information $\left\{x_{l}\left(n\right)\right\}$ from its neighbors to update the estimate $\theta_{k}\left(n\right)$; while in the combination step, each CU combines the estimates from its neighbors to obtain the intermediate estimate $P_{k}\left(n+1\right)$.

For less information communications, we set A=I. No information exchange is performed in the first part, so the ATC DMCC (19) reduces to:

(20) $$\left\{\begin{array}{c} \theta_{k}\left(n\right)=P_{k}\left(n\right)+\mu_{k}\exp\left(-\frac{1}{2\sigma^{2}}{\left|{\left|x_{k}\left(n\right)\right|}^{2}-P_{k}\left(n\right)\right|}^{2}\right)\left({\left|x_{k}\left(n\right)\right|}^{2}-P_{k}\left(n\right)\right)\\ P_{k}\left(n+1\right)=\sum_{l\in N_{k}}\gamma_{l,k}\theta_{l}\left(n\right) \end{array}\right.$$

### 4.3. ATC DMCC-Based Robust Spectrum Sensing

We summarize (20) together with energy detection as Algorithm 1.
**Algorithm 1** ATC DMCC-Based Robust Spectrum Sensing.Start with $P_{k}\left(0\right)=P\left(0\right)$ for each CU. Choose proper coefficients $\gamma_{l,k}$, μ and σ.for every time instant n≥1 do  for every CU k=1,…,K, do    1. Power estimation:       Adaption:       $\theta_{k}\left(n\right)=P_{k}\left(n\right)+\mu_{k}\exp\left(-\frac{1}{2\sigma^{2}}{\left|{\left|x_{k}\left(n\right)\right|}^{2}-P_{k}\left(n\right)\right|}^{2}\right)\left({\left|x_{k}\left(n\right)\right|}^{2}-P_{k}\left(n\right)\right)$       Combination:       $P_{k}\left(n+1\right)=\sum_{l\in N_{k}}\gamma_{l,k}\theta_{l}\left(n\right)$    2. Detection decision:       $H_{0}$ : $P_{k}\left(n+1\right)<\lambda$ or $H_{1}$ : $P_{k}\left(n+1\right)>\lambda$    The threshold λ is described in detail in Subsection 5.3.  end forend for


## 5. Performance Analysis

In this section, we study the performance analysis of the proposed algorithm. The mean performance and the variance of the performance of the proposed algorithm are analyzed first. Then, we study the energy detection performance. In order to facilitate the analysis, the following assumptions are to be adopted.

**Assumption** **1.** All input signals $x_{k}\left(n\right)$ are spatially and temporally independent.

**Assumption** **2.** The error nonlinearity $r_{k}\left(n\right)=\exp\left(-\frac{1}{2\sigma^{2}}{\left|{\left|x_{l}\left(n\right)\right|}^{2}-\sum_{i\in N_{k}}\beta_{k,i}P_{i}\left(n\right)\right|}^{2}\right)$ is independent of the input signal $x_{k}\left(n\right)$.

Strictly speaking, Assumption 2 does not accord with this fact because $r_{k}\left(n\right)$ is a function of error. However, this function can be considered as a variable step size term.

Because of the information exchange amongst CUs, the current estimates will affect their update. Therefore, in view of this dependence between CUs, we study the performance of the whole network. The proposed DMCC algorithm can be expressed as:

(21) $$P_{k}\left(n+1\right)=\sum_{l\in N_{k}}\beta_{k,l}P_{l}\left(n\right)+\mu_{k}\sum_{i\in N_{k}}\alpha_{l,k}r_{k}\left(n\right)\left({\left|x_{l}\left(n\right)\right|}^{2}-\sum_{l\in N_{k}}\beta_{k,l}P_{l}\left(n\right)\right)$$

When A=I, the algorithm will reduce to a simple version as:

(22) $$P_{k}\left(n+1\right)=\sum_{l\in N_{k}}\beta_{k,l}P_{l}\left(n\right)+\rho_{k}\left(n\right)\left({\left|x_{k}\left(n\right)\right|}^{2}-\sum_{l\in N_{k}}\beta_{k,l}P_{l}\left(n\right)\right)$$

where $\rho_{k}\left(n\right)=\mu_{k}r_{k}\left(n\right)$ as a new step size factor.

Furthermore, for more convenience, some other new variables are needed. Then, we stack the local ones into global variables as follows:

(23) $$\begin{array}{c} P\left(n\right)=col\left\{P_{1}\left(n\right),P_{2}\left(n\right),\cdots ,P_{K}\left(n\right)\right\} \end{array}$$

(24) $$\begin{array}{c} \Psi \left(n\right)=diag\left\{\rho_{1}\left(n\right),\rho_{2}\left(n\right),\cdots ,\rho_{K}\left(n\right)\right\} \end{array}$$

(25) $$\begin{array}{c} X\left(n\right)=col\left\{{\left|x_{1}\left(n\right)\right|}^{2},{\left|x_{2}\left(n\right)\right|}^{2},\cdots ,{\left|x_{K}\left(n\right)\right|}^{2}\right\} \end{array}$$

We define extra matrix U, which contains the step size parameters as follows

(26) $$\begin{array}{c} U=diag\left(\mu_{1},\mu_{2},\cdots ,\mu_{K},\right) \end{array}$$

According to the above new variables above, we remodel the update equations to represent the global network:

(27) $$\begin{array}{c} P\left(n+1\right)=BP\left(n\right)+\Psi \left(n\right)\left[X\left(n\right)-BP\left(n\right)\right] \end{array}$$

where $\Psi \left(n\right)=UR\left(n\right)$ is a diagonal matrix, and $R\left(n\right)$ is defined by:

(28) $$\begin{array}{c} R\left(n\right)=diag\left\{\exp\left(-\frac{1}{2\sigma^{2}}{\left|{\left|x_{1}\left(n\right)\right|}^{2}-\sum_{l\in N_{1}}\beta_{1,l}P_{l}\left(n\right)\right|}^{2}\right),\exp\left(-\frac{1}{2\sigma^{2}}{\left|{\left|x_{2}\left(n\right)\right|}^{2}-\sum_{l\in N_{2}}\beta_{2,l}P_{l}\left(n\right)\right|}^{2}\right)\right.\\ \left.,\cdots ,\exp\left(-\frac{1}{2\sigma^{2}}{\left|{\left|x_{K}\left(n\right)\right|}^{2}-\sum_{l\in N_{K}}\beta_{K,l}P_{l}\left(n\right)\right|}^{2}\right)\right\} \end{array}$$

Through the above equations, we can derive the mean performance and the variance performance.

### 5.1. Mean Performance

According to (27), we can rewrite the recursion as follows:

(29) $$\begin{array}{c} P\left(n+1\right)=\left[I-\Psi \left(n\right)\right]BP\left(n\right)+\Psi \left(n\right)X\left(n\right) \end{array}$$

Taking the expectation on both sides of (30), we have:

(30) $$\begin{array}{c} E\left[P\left(n+1\right)\right]=E\left[I-\Psi \left(n\right)\right]BE\left[P\left(n\right)\right]+E\left[\Psi \left(n\right)X\left(n\right)\right] \end{array}$$

We employ Assumption 2 to infer that the matrix $\Psi \left(n\right)$ is independent of the matrix $X\left(n\right)$, and then, we have:

(31) $$\begin{array}{c} E\left[P\left(n+1\right)\right]=B\left[I-E\left[\Psi \left(n\right)\right]\right]E\left[P\left(n\right)\right]+E\left[\Psi \left(n\right)\right]E\left[X\left(n\right)\right] \end{array}$$

From (31), the mean is stable if and only if the eigenvalues of matrix $B\left[I-E\left[\Psi \left(n\right)\right]\right]$ satisfy the following condition:

(32) $$\begin{array}{c} \left|\lambda_{\max}\left(B\left[I-E\left[\Psi \left(n\right)\right]\right]\right)\right|=\left|\lambda_{\max}\left(BZ\right)\right|<1 \end{array}$$

where $Z=I-E\left[\Psi \left(n\right)\right]$, and the maximum eigenvalue of a matrix is denoted by $\lambda_{\max}\left(\cdot \right)$. Because of the relation $∥BZ∥\leq ∥B∥∥Z∥$ and $∥B∥=1$, we derive $\left|\lambda_{\max}\left(BZ\right)\right|\leq \left|\lambda_{\max}\left(Z\right)\right|$. The algorithm will be stable if $\left|\lambda_{\max}\left(Z\right)\right|<1$, so we have:

(33) $$\begin{array}{c} \left|\lambda_{\max}\left(Z\right)\right|=\left|\lambda_{\max}\left(I-E\left[\Psi \left(n\right)\right]\right)\right|=\left|\lambda_{\max}\left(I-\mu_{k}E\left[R\left(n\right)\right]\right)\right|<1 \end{array}$$

Thus, the step size satisfies:

(34) $$\begin{array}{c} \left|1-\mu_{k}E\left[r_{k}\left(n\right)\right]\right|<1 \end{array}$$

We further derive:

(35) $$\begin{array}{c} 0<\mu_{k}<\frac{2}{E\left[r_{k}\left(n\right)\right]},k=1,2,\cdots ,K \end{array}$$

Therefore, the algorithm will be stable if the step size is in the bound of (35).

It is necessary to note that the condition of (35) is similar to those in [17,18]; the only difference is the extra term $E\left[r_{k}\left(n\right)\right]$, which is the expectation of the error nonlinearity introduced by MCC.

### 5.2. Variance Performance

We denote the covariance of the estimate as $Cov\left(P\left(n\right)\right)$, which is defined as:

(36) $$\begin{array}{c} Cov\left(P\left(n\right)\right)=E\left[{∥P\left(n\right)-E\left[P\left(n\right)\right]∥}_{\Sigma }^{2}\right] \end{array}$$

Substituting (29) and (30) into (36), we have:

(37) $$\begin{array}{cc} Cov\left(P\left(n+1\right)\right)= & E\left[∥\left[I-\Psi \left(n\right)\right]XP\left(n\right)+\Psi \left(n\right)X\left(n\right)-\right.\\ & \left.{\left[I-E\left[\Psi \left(n\right)\right]\right]BE\left[P\left(n\right)\right]-E\left[\Psi \left(n\right)\right]E\left[X\left(n\right)\right]∥}_{\Sigma }^{2}\right] \end{array}$$

We can see that when the kernel size is large, the elements in $\Psi \left(n\right)=\mu_{k}R\left(n\right)$ are very small, and the variation of $\Psi \left(n\right)$ is also very small. Therefore, we can consider that $\Psi \left(n\right)=E\left[\Psi \left(n\right)\right]$. Thus, we have:

(38) $$\begin{array}{cc} Cov\left(P\left(n+1\right)\right)= & E\left[∥B\left(I-E\left[\Psi \left(n\right)\right]\right)\left(P\left(n\right)-E\left[P\left(n\right)\right]\right)+\right.\\ & \left.{E\left[\Psi \left(n\right)\right]\left(X\left(n\right)-E\left[X\left(n\right)\right]\right)∥}_{\Sigma }^{2}\right] \end{array}$$

Considering the fact that $P\left(n\right)$ and observation vector $X\left(n\right)$ are independent, the covariance recursion can be shown as:

(39) $$\begin{array}{cc} Cov\left(P\left(n+1\right)\right)= & B\left(I-E\left[\Psi \left(n\right)\right]\right)E\left[P\left(n\right)-E\left[P\left(n\right)\right]\right]\left(I-E\left[\Psi \left(n\right)\right]\right)B^{T}+\\ & E\left[\Psi \left(n\right)\right]E\left[X\left(n\right)-E\left[X\left(n\right)\right]\right]E\left[\Psi \left(n\right)\right] \end{array}$$

Therefore, we have:

(40) $$\begin{array}{cc} Cov\left(P\left(n+1\right)\right)= & B\left(I-E\left[\Psi \left(n\right)\right]\right)Cov\left(P\left(n\right)\right)\left(I-E\left[\Psi \left(n\right)\right]\right)B^{T}+\\ & E\left[\Psi \left(n\right)\right]Cov\left(X\left(n\right)\right)E\left[\Psi \left(n\right)\right] \end{array}$$

This is the transient behavior of the network. Although (40) does not explicitly show the variance performance, it is in fact subsumed in the weighting matrix $B\left(I-E\left[\Psi \left(n\right)\right]\right)$, which varies for each iteration. However, the effect of the algorithm on the performance is clearly shown in (40).

### 5.3. Detection Performance Analysis

In order to derive the probability of false alarm ($P_{f}$) and detection ($P_{d}$) for the proposed algorithm, the probability density function (PDF) of the test statistic $P_{k}\left(n\right)$ under both hypotheses $H_{0}$ and $H_{1}$ needs to be evaluated.

**Figure 1:**
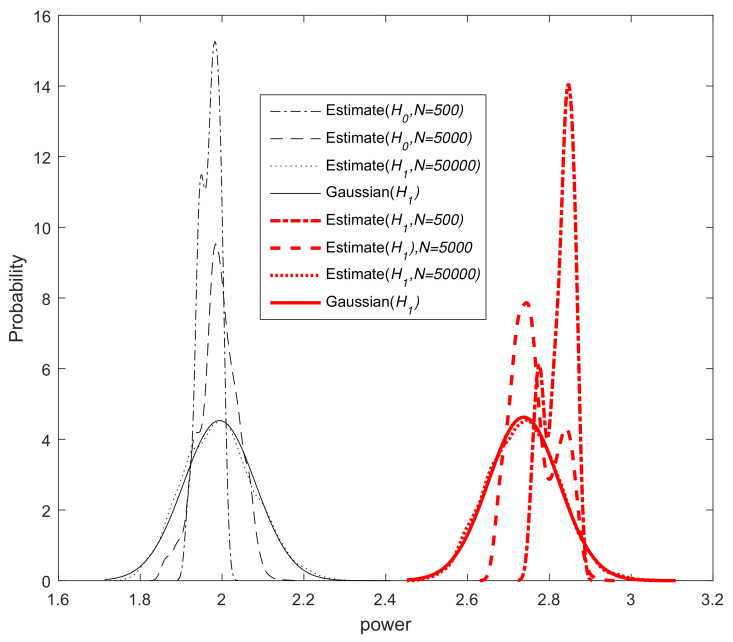
PDF of the power estimation and the Gaussian distribution.

As mentioned earlier, the DMCC method is very robust to outlier points. Thus, when an impulsive noise occurs, the factor $\exp\left(-\frac{1}{2\sigma^{2}}{\left|{\left|x_{k}\left(n\right)\right|}^{2}-\Psi_{k}\left(n\right)\right|}^{2}\right)$, which can be seen as the weight of the error ${\left|x_{k}\left(n\right)\right|}^{2}-\Psi_{k}\left(n\right)$, is close to zero, so the impulsive noise does not lead to a large estimate. Therefore, when K=1, we can regard the test statistic of energy detection $P_{k}\left(n\right)$ as a chi-square distributed random variable with 2N degrees of freedom. The test statistic $P_{k}\left(n\right)$ consists of a lot of identically distributed variables. According to the central limit theorem (CLT), when the number of samples is large enough, the chi-square distribution is approximated by a Gaussian distribution [35]. However, when K>1, the test statistic $P_{k}\left(n\right)$ in the case of hypothesis $H_{1}$ consists of a sum of a various independent, but not identically distributed variables. In this case, we apply the Lyapunov CLT [36]; when the number of samples *N* is large enough, $P_{k}\left(n\right)$ is a Gaussian approximation. The simulation result (Figure 1), shows that this approximation is reasonable. In this case, when $P_{k}\left(n\right)$ is a Gaussian approximation, the formulas for the $P_{f}$ and $P_{d}$ of the energy detector can be derived under the Neyman–Pearson criterion [37].

By taking the previous results into account, the approximate formulas for the recursive performance are derived. The probability of false alarm $P_{f}$ under hypothesis $H_{0}$ is given as follows:

(41) $$\begin{array}{c} P_{f}=Q\left(\frac{\lambda -E\left(\left.P_{k}\left(n\right)\right|H_{0}\right)}{\sqrt{Var\left(\left.P_{k}\left(n\right)\right|H_{0}\right)}}\right) \end{array}$$

where λ is the threshold, and $Q\left(x\right)=\frac{1}{\sqrt{2\pi }}\int_{x}^{\infty }\exp\left(-\frac{t^{2}}{2}\right)dt$.

Similarly, the probability of detection $P_{d}$ under hypothesis $H_{1}$ is given as follows:

(42) $$\begin{array}{c} P_{d}=Q\left(\frac{\lambda -E\left(\left.P_{k}\left(n\right)\right|H_{1}\right)}{\sqrt{Var\left(\left.P_{k}\left(n\right)\right|H_{1}\right)}}\right) \end{array}$$

The sensing threshold of the detector can be calculated from (41) by the predefined value of $P_{f}$. Thus:

(43) $$\begin{array}{c} \lambda =Q^{-1}\left(P_{f}\right)\sqrt{Var\left(\left.P_{k}\left(n\right)\right|H_{0}\right)}+E\left(\left.P_{k}\left(n\right)\right|H_{0}\right) \end{array}$$

The threshold is determined by the noise power and the predefined false alarm probability $P_{f}$.

## 6. Simulation Results

**Figure 2:**
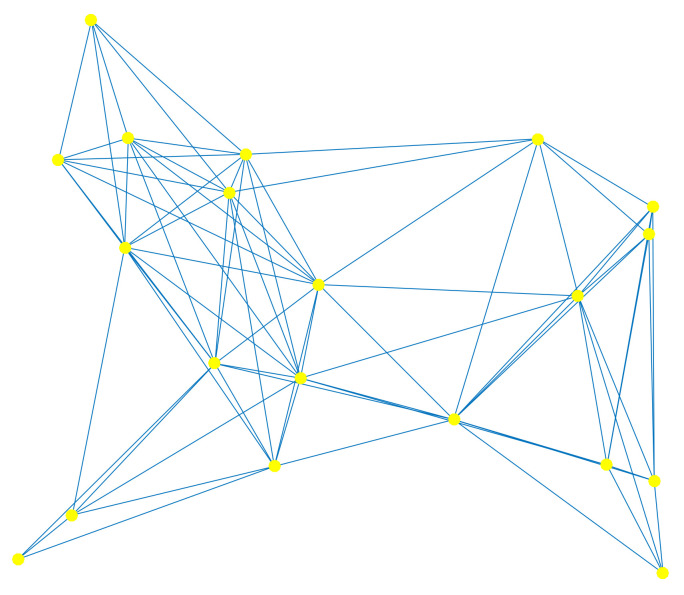
Network topology with K=20 cognitive users (CUs).

In this section, the performance of the proposed algorithm is simulated. In order to verify the performance, we consider a distributed network with K=20 CUs. The network topology is generated as the realization of the random geometric graph model (shown in Figure 2). The parameters for measurement Gaussian mixture noise are $\sigma_{1}^{2}=1$, $\sigma_{2}^{2}=100\sigma_{1}^{2}$ and $P_{r}\left(d_{k}\left(n\right)=1\right)=p=0.01$. We examine the learning performance of the new algorithm by the global average mean-square error, $MSE=\frac{1}{K}\sum_{k=1}^{K}E\left[{\left|P^{0}-P_{k}\left(n\right)\right|}^{2}\right]$. We compare the performance of the new power estimation algorithm with the diffusion algorithm called ATC DLMS [18].

### 6.1. Performance of Power Estimation Comparison among the New Method and Other Algorithms

First of all, we investigate the power estimation algorithm and the comparison among the new method (ATC DMCC) and ATC DLMS in [18]. The channel gain is assumed to be constant and generated by a standard normal distribution: $a_{k}∼N\left(0,1\right)$. All algorithms use the same channel gain. To guarantee the almost same initial convergence rate, we set the step sizes at 0.001 and 0.0004 for the mentioned ATC DMCC and ATC DLMS, respectively. The kernel size is chosen as 50 for ATC DMCC algorithm. Furthermore, all the CUs receive $N=4\times 10^{4}$ samples, and the PU signal is absent during the first half of the samples $n=1,2,\ldots ,2\times 10^{4}$ and present in the other half of samples range. Under both detection hypotheses, all CUs are disturbed by the same Gaussian mixture noise. The combination weights $\gamma_{l,k}$ are calculated by the averaging rule: [38]

(44) $$\gamma_{l,k}=\left\{\begin{array}{c} \frac{1}{n_{k}},\;\mathrm{if}\;k\neq l\;\mathrm{are}\;\mathrm{neighbors}\;\mathrm{or}\;k=l\\ 0,\;\;\;\mathrm{otherwise} \end{array}\right.$$

where $n_{k}$ is the degree of CU *k*, which means CU *k* has $n_{k}$ neighbors. All parameters are set by scanning for the best results. All the simulation results are obtained by taking the ensemble average of the network over 100 independent Monte Carlo runs.

**Figure 3:**
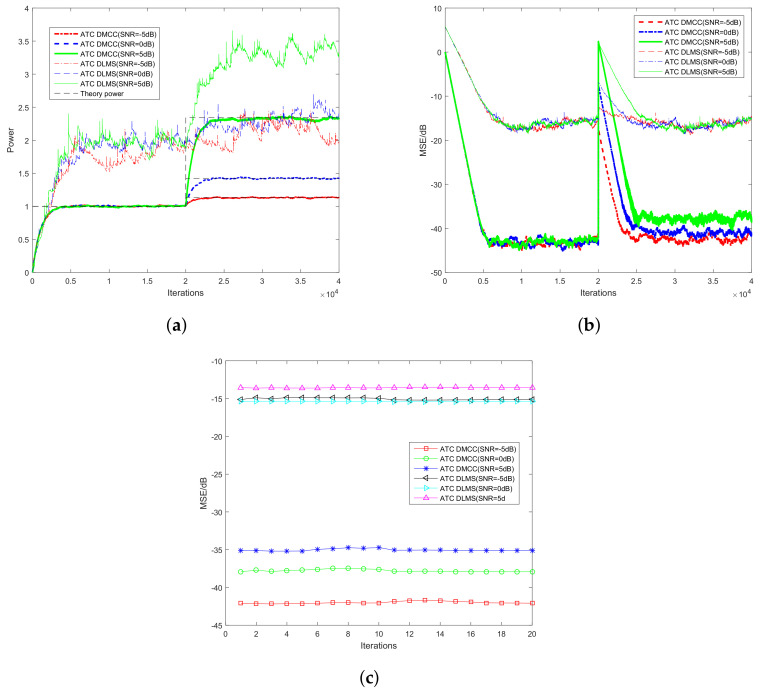
Performance in terms of power estimation with different SNRs. ATC, adaptation to combination; DMCC, diffusion maximum correntropy criterion. (**a**) Convergence curves in terms of power estimation; (**b**) Convergence curves in terms of MSE; (**c**) MSE at steady-state for the 20th CU.

Figure 3 shows the performance curves in terms of power estimation with different signal to noise ratios (SNR = −5 dB, 0 dB, 5 dB). Figure 3a shows the convergence curves in terms of the power estimation. One can observe that the ATC DMCC algorithm works well in Gaussian mixture noise, while the ATC DLMS algorithm fluctuates significantly. As one can see from the results, the proposed ATC DMCC algorithm has a much better performance in convergence rate and accuracy compared with the ATC DLMS algorithm. The results confirm that the proposed algorithm shows a significant improvement in robust performance in impulsive noise environments. Figure 3b shows the convergence curves in terms of MSE, and Figure 3c shows the steady-state MSEs at each CU *k*. As expected, the ATC DMCC algorithm performs better than the ATC DLMS algorithm.

**Figure 4:**
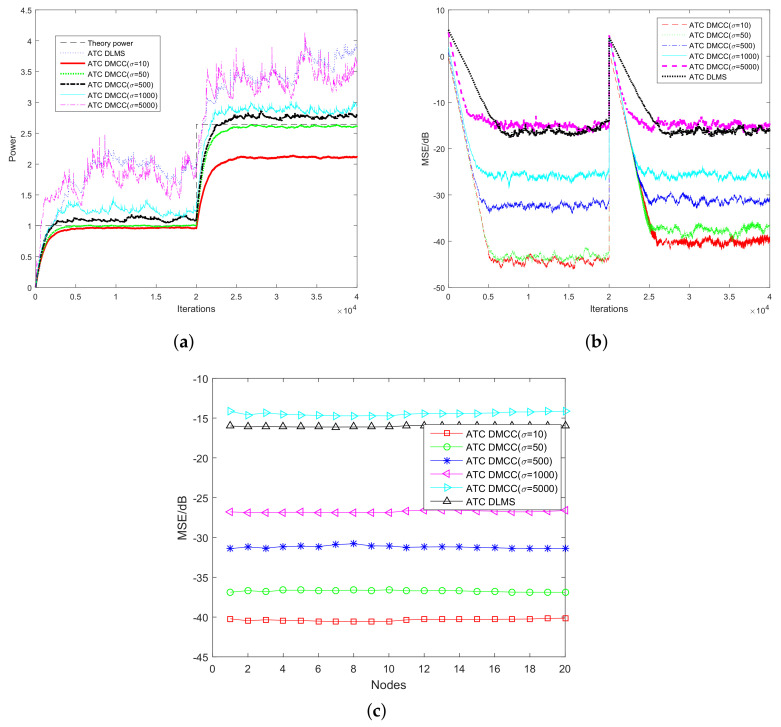
Performance in terms of power estimation with different σ. (**a**) Convergence curves in terms of power estimation; (**b**) Convergence curves in terms of MSE; (**c**) MSE at steady-state for the 20th CU.

Secondly, we compare the learning performance of the ATC DLMS algorithm with that of the proposed ATC DMCC under different kernel sizes σ. The kernel sizes of ATC DMCC are selected at σ = 10, 50, 500, 1000, 5000, respectively. The SNR is chosen as 5 dB. The other parameters for the algorithms keep the same as those in the previous simulation. It is known that kernel size is a key parameter for the proposed ATC DMCC algorithm. When the kernel size is σ→∞, the ATC DMCC algorithm degenerates into an ATC DLMS algorithm whose robustness is poor. From Figure 4, we can see that when the kernel size σ=5000 (which is considered to be pretty large), the power estimation of the proposed ATC DMCC is as poor as ATC DLMS. On the other hand, the smaller the kernel size we choose, the stronger robustness is obtained. However, the difference between power estimates (being absent or present under the PU signal) is smaller, as well. This is because if the kernel size is small, the scaling factor $\exp\left(-\frac{1}{2\sigma^{2}}{\left|{\left|x_{l}\left(n\right)\right|}^{2}-\psi_{k}\left(n\right)\right|}^{2}\right)$ in (17) will suppress the impulsive noise more effectively; the power estimate on the other hand will also be small. After consideration of the difference between power estimations (under $H_{0}$ and $H_{1}$) and the robustness of the proposed ATC DMCC, the kernel size is selected at σ=50 for the following simulations.

### 6.2. Probability of Detection Comparison among ATC DMCC and ATC DLMS

**Figure 5:**
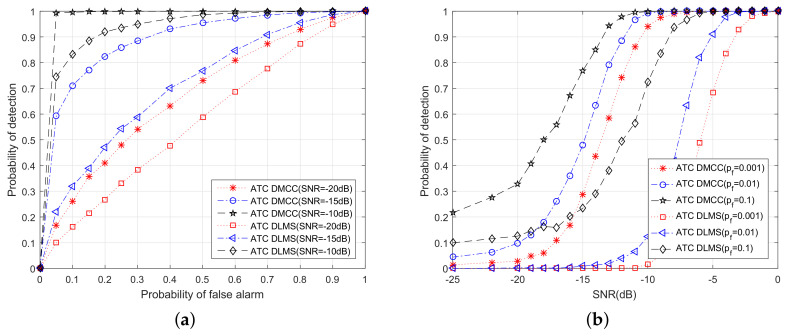
The probability of detection, ATC DMCC vs. ATC DLMS. (**a**) Description of what is contained in the first panel; (**b**) Description of what is contained in the second panel.

Next, the probability of detection of the proposed robust power estimation algorithm is investigated. Then, we simulate the comparison among the new method and ATC DLMS. In the following simulations, we also set the network size K=20 CUs. Figure 5 is the simulation result of ATC DMCC and ATC DLMS. Specifically, Figure 5a shows the receiver operating characteristic (ROC) curves of both schemes with different noise power levels (SNR = −15 dB, −10 dB, −5 dB). Figure 5b is the detection result with different false alarm probabilities ($P_{f}=0.001,0.01,0.1$). As represented in Figure 5, the proposed algorithm has a much better performance than the ATC DLMS algorithm in non-Gaussian noise. This is because of the fact that the new method has the ability to restrain the impulse noise.

### 6.3. Probability of Detection of ATC DMCC with Different Network Sizes

**Figure 6:**
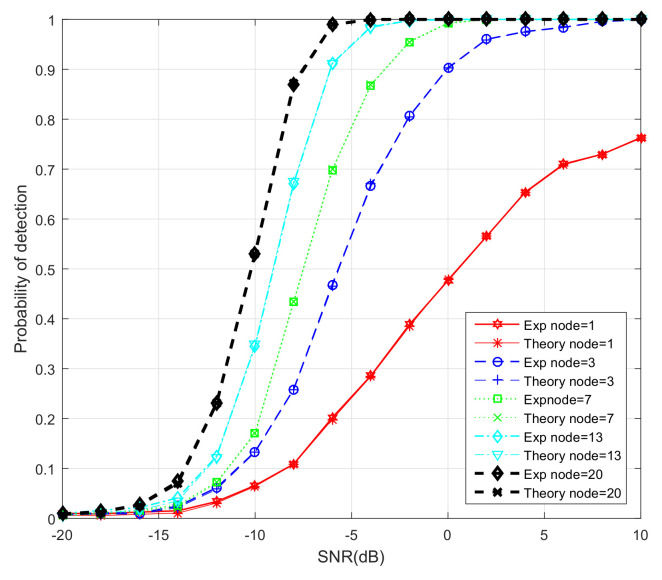
The probability of the detection of ATC DMCC with different CU numbers.

Lastly, the performances of five different network sizes (*K* = 1, 3, 7, 13, 20) are simulated. The desired probability of false alarm is chosen as $P_{f}=0.001$. In order to better display the simulation results, we set the step size at 0.004 for ATC DMCC. The comparison among the estimated and theoretical results of $P_{d}$ is simulated. In the experiment, the threshold of the energy detector is calculated by using (43) for each CU under the detection hypothesis. The theoretical $P_{d}$ is obtained by using (42). Figure 6 shows the detection performance curves of the proposed algorithm with different network sizes. One can observe that the $P_{d}$ increases as the number of CUs increases. We can also see that the detection performance is the worst when K=1. In this case, there is not much that can be done to improve the $P_{d}$ if only one CU is used to detect the spectrum. For K=3, K=7 and K=13, the detection probability increases due to the distributed estimation. However, when the network size is large enough (i.e., K=13), the $P_{d}$ increases a little as the number of CUs *K* increases. Meanwhile, we can see that the simulated and the theoretical $P_{d}$ are almost the same.

## 7. Conclusions

In this paper, we propose a version of the DMCC-based robust spectrum sensing scheme, namely the ATC DMCC algorithm, for impulsive noise. The new algorithm shows strong robustness against impulsive disturbance as MCC is very effective at handling non-Gaussian noise with large outliers. Mean and variance convergence analyses have been carried out. We also theoretically analyzed the detection performance of the new method. The performance of the proposed distributed ATC DMCC-based spectrum sensing algorithm has been compared with the ATC DLMS-based spectrum sensing [18]. Simulation results illustrate that the proposed algorithm performs very well in non-Gaussian noise environments. It can be concluded that the ATC DMCC method can achieve better performance than its predecessor (ATC DLMS) in impulsive noise.
